# Nanoarchitectonics in Materials Science: *Method for Everything in Materials Science*

**DOI:** 10.3390/ma16196367

**Published:** 2023-09-23

**Authors:** Katsuhiko Ariga, Rawil Fakhrullin

**Affiliations:** 1 Research Center for Materials Nanoarchitectonics, National Institute for Materials Science (NIMS), 1-1 Namiki, Tsukuba 305-0044, Japan; 2 Graduate School of Frontier Sciences, The University of Tokyo, 5-1-5 Kashiwa-no-ha, Kashiwa 277-8561, Japan; 3 Institute of Fundamental Medicine and Biology, Kazan Federal University, Kreml uramı 18, 42000 Kazan, Republic of Tatarstan, Russia; kazanbio@gmail.com

The history of mankind has been accompanied by the development of materials science. The quality of human life improves with the creation of more convenient materials. In prehistoric times, materials were extracted from nature and processed for use. Subsequently, the development of various scientific fields has made it possible to create desired substances. These are the contributions of organic chemistry, inorganic chemistry, polymer chemistry, coordination chemistry, supramolecular chemistry, material chemistry, and bio-related chemistry. Furthermore, it has become clear that the function of a substance can be greatly improved not only by its material but also by its internal structure. Nanotechnology has greatly paved the way for this. The development of nanotechnology has enabled mankind to directly observe and evaluate structures at the nanometer level. What is needed next is to use the knowledge of nanotechnology to assemble functional materials. As a methodology to assemble functional materials from nano-units, nanoarchitectonics was created as a post-nanotechnology [1].

Nanoarchitectonics is a methodology for assembling functional materials using nano-units such as atoms, molecules, and nanomaterials as building blocks. Nanoarchitectonics is a concept that contributes to nanotechnology and various materials science fields as mentioned above, as well as advanced technologies such as microfabrication and new emerging fields such as biotechnology. It selects from and combines atomic and molecular manipulation, chemical and physical transformation of matter, self-assembly/self-organization, orientational controls by external fields, nano- and micro-level fabrication, and biological-related technologies to construct functional materials. Because it combines several of these processes, nanoarchitectonics is well suited to create asymmetric and hierarchical structures.

Nanoarchitectonics is applicable regardless of the type of material or its function and application. Therefore, it applies to many fields, including advanced fields such as energy, environment, and medical fields, as well as basic chemistry [2,3,4,5,6,7,8]. In this Special Issue, we have collected papers related to nanoarchitectonics from this perspective. Since all materials are originally composed of atoms and molecules, nanoarchitectonics is a universal methodology to create different types of materials. In analogy to the theory of everything in physics, nanoarchitectonics can be considered as a method for any kind of material in materials science [9].
